# Supplementary material for: E-Nose Technology for Mycotoxin Detection in Feed: Ready for a Real Context in Field Application or Still an Emerging Technology?
Source: Toxins (Basel). 2023 Feb 11;15(2):146. doi: 10.3390/toxins15020146 (PMC9958648; doi:10.3390/toxins15020146)

Figure S1. Chemical structures of the mycotoxins

Aflatoxin B_1,

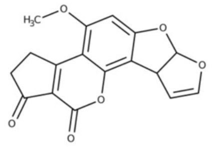

Aflatoxin B_2,

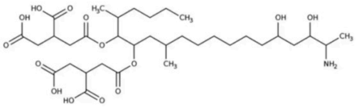

Fumonisin B_1

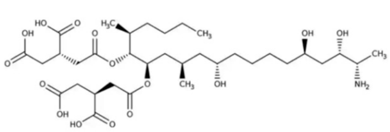

Fumonisin B_2

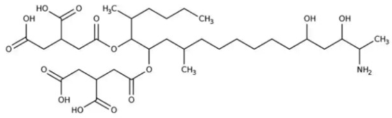

Ochratoxin

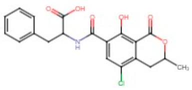

Zearalenone

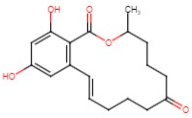

Supplement: Supplementary file 1 [file toxins-15-00146-s001.zip › toxins-2120352-supplementary.pdf]
